# Classificação da NYHA e as Variáveis do Teste de Exercício Cardiopulmonar em Pacientes com Insuficiência Cardíaca

**DOI:** 10.36660/abc.20220196

**Published:** 2022-06-06

**Authors:** Ricardo Vivacqua Cardoso Costa

**Affiliations:** 1 Hospital Pró-cardíaco Rio de Janeiro RJ Brasil Hospital Pró-cardíaco, Rio de Janeiro, RJ – Brasil

**Keywords:** Insuficiência Cardíaca, Hospitalização, Mortalidade, Consumo de Oxigênio, Teste de Esforço, Teste de Caminhada, Atividade Física, Estilo de Vida

A insuficiência cardíaca (IC) é considerada uma doença prevalente, limitando a sobrevida e constituindo uma das principais causas de hospitalização ou morte em vários países, incluindo o Brasil.^1^ Uma classificação clínica dos portadores de IC pode ser considerada importante, como referência inicial, por informar a condição funcional desses pacientes. Classicamente, são utilizadas a classificação subjetiva da New York Heart Association (NYHA) e a objetiva de Weber.^2^ A classificação funcional da NYHA e o consumo de oxigênio no pico do esforço foram determinantes na determinação da condição funcional em portadores da Doença de Chagas,^3^ porém, em outras patologias observa-se certos pacientes pouco sintomáticos e com alto risco de hospitalização ou de morte.^4^

Nos portadores de IC considera-se, também, na avaliação da capacidade funcional e prognóstica o Teste de Caminhada de 6 minutos, considerado com valor preditivo de mortalidade em pacientes com IC classe funcional II e III (NYHA).^5^

Os estudos da avaliação cardiopulmonar têm se expandido, simultaneamente, aos estudos da fisiologia do exercício, com melhor precisão na avaliação funcional e, através dos parâmetros obtidos no Teste Cardiopulmonar de Exercício (TCPE), se têm variáveis de inferência prognóstica, as quais definem condutas e orientam na prescrição de exercícios.^6^

O estudo de Ritt et al.,^7^ bem delineado, analisou correlação e concordância entre as classes da NYHA e as variáveis do TCPE. Foram destacadas as variáveis mais estudadas na atualidade.^1^ Sugerimos, como continuação do estudo, incluir correlações com a Potência Circulatória (Pressão Arterial Sistólica máxima x V’O_2_ pico)^8^ e V’O_2_ no limiar I,^9^ parâmetros que determinam perspectivas prognósticas e, como futuro estudo, o escore de risco para prever a mortalidade pós alta hospitalar em pacientes com IC.^10^

Reiteramos nossos cumprimentos aos autores^7^ pelo estudo e, pela sugestão para futuras pesquisas visando uma classificação baseada nos parâmetros obtidos no TCPE, com acurácia para indicação de transplante cardíaco ou colocação de ventrículo artificial.

## References

[1] Sociedade Brasileira de Cardiologia. Comitê Coordenador da Diretriz de Insuficiência Cardíaca. Diretriz Brasileira de Insuficiência Cardíaca Crônica e Aguda. Arq Bras Cardiol.2018; 111(3):436-539. doi: 10.5935/abc.20180190. 10.5935/abc.20180190 30379264

[2] Weber KT, Kinasewitz GT, Janick JS, Fishman AP. Oxygen utilization and ventilation during exercise in patients with chronic cardiac failure. Circulation.1982;65(6):1213-23. doi:10.1161/01.circ.65.6.1213. 10.1161/01.cir.65.6.1213 6804111

[3] Silva WT, Costa HS, Figueiredo PHS, Lima MMO, Lima VP, Costa FSM, et al. Determinantes da Capacidade Funcional em Pacientes com Doença de Chagas. Arq Bras Cardiol. 2021;117(5): 934-41. doi:https://doi.org/10.36660/abc.20200462. 10.36660/abc.20200462 PMC8682109 34378673

[4] Esc Guidelines for the diagnosis and treatment of acute and chronic heart failure. Eur Heart J.2021;42(36):3599-726. doi:10.1093/eurheartj/ehab368. 10.1093/eurheartj/ehab368 34447992

[5] Morais ER, Rassi S. Determinants of the distance covered during a six-minute walk test in patients with chronic heart failure. Int J Cardiovasc Sci. 2019;32(2):2019;32(2):134-42. doi: 10.5935/2359-4802.20180088

[6] Ávila DX, Vivacqua RC, Serra S, Montera MW, Tinoco E, Siciliano A. The unsurpassed value of cardiopulmonary exercise testing in assessing the prognosis of heart failure. Eur Heart J.2020;41(Suppl 2):ehaa946.988. https://doi.org/10.1093/ehjci/ehaa946.0988

[7] Ritt LEF, Ribeiro RS, Souza IPMA, Ramos JVSP, Ribeiro DS, Feitosa GF, et al. Baixa concordância entre a classificação da NYHA e as variáveis do teste de exercício cardiopulmonar em pacientes com insuficiência cardíaca e fração de ejeção reduzida. Arq Bras Cardiol. 2022; 118(6):1118-1123. 10.36660/abc.20210222 PMC9345141 35384966

[8] Tabet JY, Metra M, Thabut G, Logeart D, Cohen-Solal A. Prognostic value of cardiopulmonary exercise variables in chronic heart failure patients with or without beta-blocker therapy. Am J Cardiol.2006;98(4):500-3. doi: 10.1016/j.amjcard.2006.03.027. 10.1016/j.amjcard.2006.03.027 16893705

[9] Lala A, Shah KB, Lanfear E, Thibodeau JT, Palardy M, et al. Predictive value of cardiopulmonary exercise testing parameters in ambulatory advanced heart failure. J Am Coll Cardiol HF 2021;9:226-236-36. doi:https://doi.org/10.1016/j.chf.2020.11.008. 10.1016/j.jchf.2020.11.008 33549559

[10] Lei Wang, Li-Oin Wang, Mo-Li Gu, Liang Li, Chen Wang, Yun-Feng Xia. Escore de risco clínico simples para prever a mortalidade pós-alta hospitalar em pacientes chineses hospitalizados por insuficiência cardíaca. Arq Bras Cardiol.2021;117(4):615-23. doi:10.36660/abc.20200435. 10.36660/abc.20200435 PMC8528360 34406318

