# Supplementary material for: Classificação da NYHA e as Variáveis do Teste de Exercício Cardiopulmonar em Pacientes com Insuficiência Cardíaca
Source: Arq Bras Cardiol. 2022 Jun 6;118(6):1124–5. [Article in Portuguese] doi: 10.36660/abc.20220196 (PMC9345164; doi:10.36660/abc.20220196)
Supplement: Translation

# Translation: NYHA Classification and Cardiopulmonary Exercise Test Variables in Patients with Heart Failure

**Authors:** Ricardo Vivacqua Cardoso Costa

**Affiliations:** 1 Rio de Janeiro RJ Brazil Hospital Pró-cardíaco, Rio de Janeiro, RJ – Brazil

**Keywords:** Heart Failure, Hospitalization, Mortality, Oxygen Consumption, Exercise Test, Walk Test, Physical Activity, Life Style

Heart failure (HF) is considered a prevalent disease, limiting survival and constituting one of the leading causes of hospitalization or death in several countries, including Brazil.^1^ Therefore, clinical classification in patients with HF can be considered important as an initial reference, as it informs the functional condition of these patients. Classically, the subjective classification of the New York Heart Association (NYHA) and the objective classification of Weber^2^ are the most used. The NYHA functional classification and oxygen consumption at peak exertion were decisive in determining the functional condition of patients with Chagas disease.^3^ However, certain patients with minor symptoms are at high risk of hospitalization or death.^4^

In patients with HF, the 6-minute walk test is also considered in assessing functional and prognostic capacity. This test has a predictive value for mortality in patients with HF functional class II and III (NYHA).^5^

Studies of cardiopulmonary assessment have expanded, simultaneously, to studies of exercise physiology, with better precision in the functional evaluation and, through the parameters obtained in the Cardiopulmonary Exercise Test (CPET), we have prognostic inference variables, which define conducts and guide the prescription of exercises.^6^

The well-designed study by Ritt et al.^7^ analyzed the correlation and agreement between NYHA classes and CPET variables. The most studied variables today were highlighted.^1^ We suggest, as a continuation of the study, to include correlations with Circulatory Power (Maximum Systolic Blood Pressure x V’O2 peak)^8^ and V’O2 at the threshold I,^9^ parameters that determine prognostic perspectives and, as a future study, the risk score to predict post-discharge mortality in patients with HF.^10^

We reiterate our congratulations to the authors^7^ for the study and the suggestion for future research aiming at a classification based on the parameters obtained in the CPET, with accuracy for indication of heart transplantation or placement of artificial ventricle.
